# Taking Responsibility for Responsibility

**DOI:** 10.1093/phe/phz001

**Published:** 2019-02-11

**Authors:** Neil Levy

**Affiliations:** Department of Philosophy, Macquarie University and Uehiro Centre for Practical Ethics, University of Oxford

## Abstract

Governments, physicians, media and academics have all called for individuals to bear responsibility for their own health. In this article, I argue that requiring those with adverse health outcomes to bear responsibility for these outcomes is a bad basis for policy. The available evidence strongly suggests that the capacities for responsible choice, and the circumstances in which these capacities are exercised, are distributed alongside the kinds of goods we usually talk about in discussing distributive justice, and this distribution significantly explains why people make bad health choices. These facts suggest that we cannot justifiably hold them responsible for these choices. We do better to hold responsible those who determine the ways in which capacities and circumstances are distributed: they are indirectly responsible for these adverse health outcomes and possess the capacities and resources to take responsibility for these facts.

Calls for us to take responsibility for our health, and expressions of blame for those become ill, are common. While the harshest condemnation comes from the popular press, calls for us to take responsibility come from a variety of sources, including physicians themselves and even governments. In this article, I argue that these calls are unjustified. I argue that the capacities for responsibility, and the circumstances in which they are exercised, are themselves distributed: typically, agents can effectively take responsibility for their own health by adopting healthier lifestyles only if they are the beneficiaries of distributive mechanisms that allocate life chances.^1^ In this light, calls for us to take responsibility for our health are best understood as responsibility-shifting mechanisms: they serve to shift the burden from those who are best equipped to meet it to those who cannot.

Calls for us to take responsibility come from multiple sources. They are to be found in the popular press (Macrae, 2016), in the academic literature (Callahan, 2013) and in public statements from corporations (Kent, 2009). In this article, I am concerned with these calls only insofar as they might form a basis for public policy. Responsibility is already enshrined in the *NHS Constitution for England* (NHS, 2015) and underlies health policy in other countries. For example, Hungary reportedly uses adherence to dietary recommendations to exclude patients from access to some therapies (Hazell, 2012). It is with responsibility in these kinds of contexts that I am principally concerned. While the considerations I will cite have implications that are broader than exhortations to responsibility in these contexts, it is here that their implications are clearest. Responsibility is not a good basis for public policy, I will suggest, because policies should be formulated in ways that are insensitive to fine-grained differences in the capacities that underlie responsibility. While there may be individuals who might appropriately be asked to take responsibility for their health, they form too small a minority, and they are too difficult to identify, for responsibility to be a good basis for policy. Conversely, there is a large group of individuals who might appropriately be asked to take responsibility *for* responsibility. It is both fairer, and better policy, to address such demands to them, and not to those whose health suffers as a consequence of their own choices.

## Responsibility for Health

Should agents be expected to take responsibility for their health? Calls for us to do so have arisen in response to the recognition that ill-health is not something that just happens to us. Rather, early mortality and increases in morbidity are often due at least in important part to our behavior. Lifestyle factors are very significantly responsible for the global burden of disease: up to 40 per cent of premature deaths are preventable by changes to lifestyle (Yoon *et al.*, 2014). We are in the midst of what the World Health Organization (2003) described as an *obesity epidemic*, and it is widely held that obesity is a risk factor for cancer, heart disease and stroke (WHO, 2014).^2^ Accordingly, WHO has called for changes in lifestyle to halt this epidemic, as well as to reduce or eliminate other risk factors for early mortality and increased morbidity (WHO, 2014). Lack of sufficient exercise, excessive drinking and smoking all contribute to ill-health. Moreover, when disease arises, there is usually a great deal we can do to help to manage it, and patients fail surprisingly often to do these things—only about half of all prescribed doses of medication are taken by patients, for instance (Nieuwlaat *et al.*, 2014).

In the light of these facts, calls for us to take responsibility for our health therefore make *prima facie* sense (see Brown, 2013; Friesen, 2018 for discussion). Whether or not we fall ill depends, significantly, on what we do, and whether or not we recover depends, significantly, on what we do. They fall within the purview of our agency. We are, partly but significantly, *causally* responsible for our own health and wellbeing, and causal responsibility is widely held to be a necessary condition of moral responsibility (at least of the sort that will be under discussion). If we are causally responsible for some consequence, and that consequence falls within the sphere of facts that are properly moralized, then we may be morally responsible for it as well.

There are multiple senses of the phrase ‘moral responsibility’. For instance, on some accounts of moral responsibility attributions of responsibility are justified on consequentialist grounds: an agent is morally responsible for an action just in case it makes sense, on forward-looking grounds, to *hold* her morally responsible (her future behavior can be expected to improve, say, or others will be deterred by the example). In this article, I am concerned with moral responsibility in what has been called the basic desert sense (Pereboom, 2014): to say someone is morally responsible, in this sense, is to say that they *deserve* to be treated better or worse—say by being subjected to blame and condemnation—on backwards looking grounds *alone.*^3^ The basic desert sense of moral responsibility is probably the central sense. In fact, ordinary people appear to understand responsibility for actions and for consequences as referring first and foremost to this sense of term (Cushman, 2008). Certainly, it plays an important role in our legal system: while consequentialist considerations matter to our system of fines and imprisonment, for instance, most people appear to believe that serious sanctions should express our condemnation of the offense, independently of any salutary effects condemnation has (indeed, ordinary people are almost entirely insensitive to consequentialist considerations in assessing how severe a punishment should be; Carlsmith and Darley, 2008). When agents are responsible, in this sense, for wrongful actions, they are blameworthy.

Of course, it is controversial what conditions must be satisfied for agents to be morally responsible. In common with most people who work on the topic, I assume that agents can be morally responsibility only if they satisfy control and epistemic conditions (Fischer and Ravizza, 1998; Eshleman, 2014). At minimum, (a) the action or state of affairs for which the agent is supposed to be responsible must be causally sensitive to her actions, and (b) she must understand that this is true, and know how to intervene in it.^4^ Of course, there is a very rich debate on how to make these conditions more precise, which I can’t hope to do justice to here. It suffices to note that there is a near consensus, among philosophers (Bourget and Chalmers, 2014) and ordinary people (Roskies and Nichols, 2008) that most agents routinely satisfy these conditions. Ordinary people are either compatibilists (Murray and Nahmias, 2014), holding that even if our actions are determined, we may be responsible for them, or they are libertarians (Nichols and Knobe, 2007), holding that our actions are not determined; they are rarely skeptics.

Thus, if agents are responsible (in the relevant sense) for their ill-health, they might deserve to bear the consequences. They might, for example, be assigned a lower priority when it comes to the allocation of health resources. For instance, it has been suggested that those who are responsible for the fact that they need a liver transplant (due to their heavy drinking) should have a lower priority when it comes to the allocation of scarce organs than those whose develop the need through no fault of their own (Glannon, 1998). Public health care resources might be denied to those responsible for their ill-health altogether, or they might be required to contribute more to their care than those who are not responsible, or they might be given a lower standard of care (more expensive medications might be reserved for more deserving cases, for instance).

Of course, the allocation of scarce resources to those who have brought about their own ill-health might be justified on consequentialist grounds. In particular, sometimes attempts are made to justify them on the grounds that the expected benefit to someone who is likely to engage in unhealthy behavior in the future is too small to justify some expenditures of health dollars. Policies that assign responsibility in this kind of way do not rely on the basic desert sense of responsibility. However, it is plausible that intuitions about desert play a subterranean role in motivating these apparently consequentialist policies. Proposals to delay surgery for those who are obese or currently smoking are sometimes justified on these kinds of grounds, but these claims are likely false: the interventions appear to be cost effective (Shaw, 2016). It may be that these proposals pass scrutiny only because many people are eager to ensure that people get what they (putatively) deserve.^5^

One way to resist the conclusion that agents are morally responsible (in the basic desert sense) for their ill-health is to deny that these behaviors or their consequences fall within the sphere of morality. On most accounts, we cannot be morally responsible for the morally neutral or the nonmoral: an agent cannot be morally responsible for the color of their T-shirt or for scratching their ears: not unless it was reasonable to expect that someone might (say) be offended by the color, or they had an obligation not to scratch, and so on. There is a very plausible case for thinking that diet and its consequences (in particular) is moralized in ways that are inappropriate (think of ‘fat shaming’). Some of the opprobrium that attaches to those who engage in unhealthy behavior almost certainly stems from such inappropriate moralizing. Other people may have different tastes, and (for example) value sensual pleasure sufficiently to make it rational for them to take the risk of a shortened lifespan or a decrease in quality of life in the future. Nevertheless, the claim that ill-health is not a moral concern at all is implausible.

The very existence of the field of medical ethics testifies to our belief that healthcare is a moral issue. We believe, rightly, that it is morally incumbent on us to treat the ill, and health care budgets are finite. Money spent on one group of patients is not available for others. Difficult decisions about resource allocation must be made, and these are importantly moral decisions. Prima facie, at least, if people are responsible for their ill-health, they may be blamed on the grounds of imposing costs on a health system that must allocate scarce resources.^6^ Those people, however numerous they may be, who are rational in preferring to engage in unhealthy activities do not absolve themselves of responsibility for the consequences of their behavior, for themselves or for others. If anything, the opposite seems true: to the extent that they grasp that they trade off present pleasure for future costs, they are responsible for the choices and its consequences.

The claim that the allocation of healthcare resources should be sensitive to the responsibility of agents is therefore *prima facie* plausible. It is *prima facie* plausible that some agents *deserve* more than others to be allocated scarce organs (for instance). If an agent knowingly and voluntarily engages in an activity that risks imposing burdens on others, for trivial or self-interested reasons,^7^ that agent is *prima facie *responsible for the outcome and our response can rightfully take that fact into account. If you reject this claim, it is worth noting—if you judge that people cannot be responsible for their ill-health in virtue of their lifestyle-related behavior—then you are not my target, because you do not hold the view I aim to criticize: that a large class of agents can be given lower priority for healthcare in virtue of their responsibility.

There are, however, other grounds for denying that most people are responsible for their ill-health. I will argue that the capacities for responsible agency, *especially* the kind of responsible agency exercised over health in the kinds of cases mentioned above, are themselves socially distributed (along with, and in a way that is highly correlated with, other important goods and opportunities). Because they are so distributed, those who are (on average) least able to exercise them are the ones most in need of them. Holding these agents responsible for bad outcomes is, in this domain (though perhaps not others), deeply unfair as a consequence. Moreover, it serves to deflect responsibility from those individuals and institutions responsible for the unfair allocation of burdens and capacities, and who have the wherewithal to do something about them, to those to whom they are allocated and who do not.

I will argue that the best explanation for the social gradient in health is that capacities and circumstances are distributed in a way that ensures that those who face the most temptations have the least capacity to resist them. In doing so, I build upon, but substantially go beyond, Brown’s (2013) case that psychological mechanisms might explain the gradient and thereby undermine responsibility.

## The Social Determinants of Health

Let me begin with a striking fact: there is a strong correlation between chronic disease, increased risk of morbidity and early mortality, on the one hand, and socio-economic status (SES), on the other (Marmot *et al.*, 2008; Marmot, 2018). There is a social gradient in ill-health: the lower one’s level of education and income, the worse one’s health is (on average, of course). There are a variety of reasons for this correlation, and some factors fall outside the scope of facts over which individuals might reasonably be expected to exercise any significant degree of control. For instance, poorer people may live in environments that are less healthy (close to pollution-producing factories, or alongside major roads and therefore in proximity to exhaust emissions and to noise, both of which are known to contribute to heart disease; see Shah *et al.*, 2013 on pollution; Gan *et al.*, 2012 on noise). They may work in more stressful jobs, and have less opportunity to exercise because their local neighborhoods lack parks to walk in. These are factors over which it is not reasonable to expect them to exercise much control, because doing something about them requires resources they lack. Secure housing may not be available in better areas at a price they can afford, for example.

But causal factors in the social determinants of health include behaviors that might reasonably be thought to be within the sphere of control of the individual. For instance, there is a significant correlation between SES and rates of smoking in many countries (Greenhalgh *et al.*, 2015), but smoking is a voluntary behavior (of course, nicotine is highly addictive, which introduces complications with regard to the extent to which it is voluntary; nevertheless, at the very least beginning to smoke is voluntary). Similarly, in higher income countries (but not lower-income countries) obesity is negatively correlated with SES, and part of the explanation for these differences lies with diet quality (e.g. Darmon and Drewnowski, 2008). While healthier foods can be more expensive than less healthy, cost does not fully explain the differences in diet quality between high and low SES populations (Pechey and Monsivais, 2016).

We might take the evidence that appears to indicate that some, but not all, of the causal factors that underlie health differences between high and low SES groups are within the scope of their potential control to indicate something about the *extent* to which people should be held responsible, or the *scope* of justified responsibility ascriptions. That is, we might conclude, on the basis of the fact that some of the variance in health across demographic groups is the product of agential behavior, that agents are responsible just to the extent to which this is true, or responsible for those behaviors that are sufficiently agential. I will suggest that this response does not go anywhere near far enough. We should think that the group difference is not explained by behavior for which agents might appropriately be held responsible: though some of the social determinants of health are mediated by agential behavior, those agents with worse outcomes have (on average) significantly worse capacities to exercise what is sometimes called ‘responsibility-level’ control (Haji, 2012).

Social science aim to identify the factors that together explain outcomes. In this case, the outcome we are attempting to explain is the difference in health between high and low SES populations. We have seen that some of the variance in this difference is explained by factors over which low SES individuals (that is, those people whose health is, on average, worse) have insufficient control to justify holding them responsible. But other factors fall within the scope of their agency: they could eat better, for example. We might therefore conclude that they are somewhat responsible for their ill-health, because it is partially the result of actions of theirs for which they are responsible.

But while it might be true that agential behavior plays a role in explaining health outcomes, these behavioral differences *themselves* cry out for explanation. What explains the fact that members of one group make worse choices than members of another? Citing their free choices is no explanation at all, because it leaves entirely mysterious why there are systematic differences between groups in how choice is exercised.^8^ We should think that systematic differences like this can themselves be explained, and we should be open to the possibility that this explanation might be responsibility-undermining (or shifting).

Why would one group of individuals make choices that are worse than another? While there is no general answer to that question, in the context of these choices there are several different factors which together help to explain differences in behavior. Agents face different choices, in different contexts, with different capacities and with different senses of their options and their significance. Looking to the choices that high and low SES agents face, the contexts in which they face them, the capacities for choice they have and the senses they are likely to have of their options together go a long way toward explaining the differences in the choices they make.

I will begin with the capacities agents have. Agential behavior depends on capacities to resist impulses, to plan and to implement these plans. These are capacities that differ across groups. SES affects our brains, as much as it affects our environment (it affects our brains *by* affecting our environments). Most relevantly for our purposes, differences in working memory (the capacity to keep information in mind for a short period of time) and in inhibitory control (the capacity to resist temptations, or to inhibit habituated responses) emerge early in childhood (Lipina *et al.*, 2005; Lipina 2014). These differences increase over development, and implicate other capacities, such as the capacity to focus attention (see Hackman *et al.*, 2010 for review). In every case, lower SES correlates with reduced executive function. These differences in capacity to attend, to resist distraction, to plan and to inhibit impulses are not themselves differences over which agents have control. They develop early, before the person is in a position to make responsible choices. Together, they explain much of the difference in the choices lower SES individuals make, compared to higher.

These differences can be themselves be explained. In part, these differences are adaptive responses to the environment in which lower SES individuals find themselves. Take the capacity to inhibit impulses. This is a capacity that develops through use, and lower SES individuals get fewer opportunities to practice the capacity for inhibition. They face fewer contexts in which they will be rewarded for delaying gratification. Delay of gratification is adaptive in environments that are richer in resources, because in such environments foregoing a reward is not likely to be costly. There will be other opportunities to secure equally valuable goods. But in poorer environments, rewards foregone might be lost forever (Kidd *et al.*, 2012). We are likely sensitive to cues of resource richness, and respond by up- or downregulating mechanisms for inhibitory control (Levy, 2016). It is in part because we face choices in different contexts that we have different capacities for choice.

Whereas some of these differences represent adaptive responses to contexts, others might be explained in other ways. For instance, some of the observed differences in attentional control are the product of exposure to chronic stress (Liston *et al.*, 2009). Poverty causes stress in a variety of ways: worrying about paying bills, about the security of housing, and so on. But stress does not only undermine capacities: it also makes certain choices more tempting. Part of the reason why lower SES individuals smoke at higher rates, for instance, is that smoking alleviates stress, at least in the short term (McClernon and Gilbert, 2010). Similarly, lower SES individuals may eat tempting but unhealthy foods, in part, to alleviate stress (Adam and Epel, 2007).

Finally, SES correlates with education and therefore with knowledge of which foods are healthy and the long-term consequences of bad food choices. Thus lower SES individuals face more difficult choices—unhealthy foods are likely to be more tempting for them—with reduced capacities for making such choices, in contexts in which they are likely to see less reason for making such choices. Making worse choices is therefore not mysterious at all: it is the expected upshots of these differences in capacities, contexts and knowledge.

One might object these differences do not show that lower SES individuals are not responsible for their lifestyle-related choices. They are responding *rationally* to the circumstances in which they find themselves, and rational choice is (other things equal) responsible choice.^9^ Indeed, this point—that responsibility should be understood as centrally involving the capacity to respond, appropriately, to reasons—is at the heart of the reasons-responsiveness account to which many philosophers subscribe (Fischer and Ravizza, 1998 is the locus classicus; I have endorsed a variant of this account in Levy, 2017).

In response to this objection, several things should be emphasized. First, a significant part of the explanation for the choices of lower SES individuals involves mechanisms that are not reasons responsive. A decreased capacity to inhibit impulses is a volitional defect, rather than a cognitive mechanism. Second, the fact that the explanation for the set up of some mechanism cites reasons does not show that the mechanism is, in the relevant sense, reasons-responsive. Evolutionary theorists distinguish *distal *and *proximal* explanation. Distal explanations explain how a mechanism is adaptive: how it functions to increase the organism’s fitness. Proximal explanation explain how it is implemented. In many cases, a distal explanation cites reasons, but those reasons need not be reasons *from the organism’s perspective.* Thus, the distal explanation for decreased sensitivity to longer term rewards cites reasons: it is adaptive for organisms to prefer immediately available rewards under many conditions. But these reasons may not be reasons *for the person.* Indeed, she may recognize that in her current environment (which differs so dramatically from the environment for which these mechanisms are adaptations), she has better reason to abstain than to consume, but these reasons have reduced motivational power for her due the ways in which these mechanisms are configured.

Finally, and most importantly, the reasons-responsiveness account of moral responsibility should not be understood as simply asserting that agents are responsible for actions when they act on reasons-responsive mechanisms. Rather, the account is more fine-grained than that: an agent is responsible *for violating certain norms* if she acted on a mechanism that is responsive to the set of reasons that apply in the domain of those norms. Thus, non-human animals are arguably reasons-responsive—their behavior is guided by states of affairs that function as reasons for them—but they are not morally responsible because they lack the capacity to respond to moral reasons specifically. Analogously, it would be a mistake to conclude on the basis of the fact that their lifestyle behaviors are often guided by reasons that lower SES individuals may be held responsible for the outcomes in the kinds of way that are relevant here. It is because they have reduced capacities to respond *to the relevant reasons* that they find it difficult to guide their behavior in their light.

None of this entails that lower SES individuals are not capable of responsible choice. Typically, theorists of moral responsibility hold that agents are morally responsible for their behavior if they have *enough* in the way of the relevant capacities to make the relevant choice in the relevant context, and in addition satisfy the epistemic condition on choice sufficiently well. Particularly in the context of criminal behavior, this kind of approach has a great deal to recommend it. While we may want our justice tempered by a mercy that stems from a recognition that some people find it harder to resist temptations to criminal activity or are more likely to behave impulsively than others, most people think that above a certain threshold of capacity, it is reasonable to expect individuals to refrain from seriously immoral actions. Given the stakes, we expect normal individuals to be sufficiently motivated to marshal the resources they need to avoid such behavior.

However, in the much lower stakes contexts with which we are concerned here, the repeated context of choice of one food over another, say, the demand that people somehow find the wherewithal to make the right choices sufficiently often is much less reasonable. There are at least two reasons why it is reasonable to hold people to higher standards in higher-stakes contexts. One is that we reasonably expect that recognition that one is in a high-stakes context is motivating. When someone recognizes that something of great significance is on the line, we expect them to pay attention, to make a great effort, and so on. Thus, we accept ‘it was too hard’ as an excuse for not bringing in the washing much more easily than we do for not feeding the children, for instance. Second, high-stakes contexts, and thus the need to make an effort and attend, are relatively rare for most of us. If a particular challenge arises repeatedly, we may expect that someone will fail eventually, due to inattention, fatigue or sheer bad luck. But they have no such excuse available when the challenge is rare and high-stakes.

We might bring this out by comparing two kinds of contexts. Most of us have experienced situations in which our executive capacities are impaired (due to tiredness or alcohol consumption, say) and we have experienced a fleeting temptation to engage in clearly immoral behavior. The temptation might be to drive while seriously drunk, or to steal someone’s wallet, or even to commit more seriously wrong actions. Most of us have not engaged in the behavior, I hope: even in our impaired state, the high stakes have been sufficient to bring us to pull ourselves together and refrain. But, we cannot say the same thing about much lower stakes contexts in which we have been impaired in executive control and faced temptation. In these contexts, we may have engaged in trivially immoral behavior (not paying for a drink, or insulting someone, say) or prudentially unwise behavior (smoking, drinking more than we should, taking risks that are unjustified). These latter slips seem explicable and forgivable. But low SES individuals find themselves in analogous situations *routinely.* If our lapses are forgivable—not the kind of thing on which it makes sense to hang serious consequences—then so, it seems, are theirs. Whereas we, with our greater capacity to control our behavior in the light of (the relevant) reasons may deserve responsibility if we repeatedly engage in risky or indulgent behavior in these (individually) low stakes context, and therefore be responsible for the outcomes, lower SES individuals may not be responsible for the overall pattern of behavior and therefore for the outcomes, despite the fact that they have the wherewithal to guide their behavior by moral norms in higher-stakes contexts.

If the between-group differences are explained by these factors—factors concerning which agents have no choice, and factors which excuse their choices—then it seems we cannot rightly hold agents responsible for the consequences of their choices. Those low SES individuals who end up with poorer health as a consequence of their behavior will be those who have made poor choices often enough (not necessarily on every occasion, of course; for many such individuals, there will be many instances of successful self-control—but it takes only one slip to render many such instances otiose), and these poor choices are not such that we can reasonably expect them to make better choices.^10^

Of course, many low SES individuals do not exhibit the reduction in executive function characteristic of the group. Some will even have superior executive function. Similarly, many will not face greater temptations to engage in unhealthy behaviors, or will not experience more stressors, or more chronic stress, than those individuals in higher SES groups (equally, we will find individuals in the latter group who exhibit these deficits, are subject to these temptations, experience these stressors). We will even find individuals who suffer from *none* of these problems, internal or external. Many of these individuals will put their good fortune to good use, and engage in healthier behaviors than is typical for their group, but some will choose unhealthy behaviors, and some of this group will suffer adverse health consequences as a result. These individuals do not have the excuses that group membership makes available to others, and therefore might appropriately be held responsible (for all that has been said here).

But in this context, policy is better formulated in ways that are insensitive to these kinds of differences. For reasons of cost and efficiency, we shouldn’t subject individuals to extensive neuropsychological testing and take detailed life histories. While policy in this domain may take individual circumstances into account, it should do so more in the kind of way actuarial tables do: by considering basic demographic information, rather than fine-grained details of individual differences. It will often be difficult to discern when individuals are exceptions to the generalizations we can draw from this kind of information. We have sophisticated tests for cognitive control (e.g. Go/No-go tasks), but they are time consuming and resource intensive, and much more reliable at detecting group than individual differences. Moreover, there are good reasons to think that there is little point in enquiring into such details. Health policies that require individuals to bear responsibility for their own behavior, when it results in adverse outcomes *and* they possess, or possessed at relevant times, unimpaired executive function in propitious circumstances, would apply to a small group of individuals. Most of the people who are causally responsible for ill-health do not satisfy these conditions. Since such a policy would apply to relatively few individuals, the costs of implementing it—requiring, as it would, testing of a large number of individuals to identify the few—would likely be significantly greater than the savings in health care costs. Unless we think that wreaking retribution on these few is a high priority, such an approach is bad policy.

## Taking Responsibility for Responsibility

In the previous section, I argued that the correlation between low SES and adverse health outcomes, to the extent it is mediated by agential behavior, is very largely explained by the decreased capacities of members of that group, combined with the more demanding context in which they find themselves. Low SES individuals typically must make choices that are more difficult *and* have reduced capacities to make these choices. While their capacities may be sufficient for the kinds of challenges that fall within the scope of criminal responsibility, the combination of difficult circumstances, reduced capacities and repeated challenges to them makes it hard to hold them responsible for outcomes in the domain of health. We would do well to avoid baking assumptions about the responsibility of those whose ill-health arise from lifestyle into our health-related policies.

That is not to say, however, that responsibility is not important from a policy perspective. Policy should strive for efficient and ethical uses of scarce resources, by ensuring that individuals and institutions responsible for outcomes are held to account for them. There are appropriate targets of responsibility ascription. They are the institutions—political, judicial and corporate—and individuals actually responsible for the distribution of responsibility-relevant capacities and the distribution of the circumstances in which choices are made. Exactly how these institutions can be held responsible is a difficult question, of course. There is considerable debate in the literature over whether there are legitimate ascriptions of ‘corporate responsibility’ (Sverdlik, 1987; Sepinwall, 2016), or whether all such ascriptions are reducible to conjunctions about claims about individuals (Giubilini and Levy, 2018). However that debate is settled, responsibility surely attaches to many individuals: to policy makers, legislators, highly placed businesspeople and perhaps ordinary people (especially higher SES individuals) in their capacity as voters.

The choices of these individuals and institutions play a significant role in the distribution of the capacities other individuals find themselves with. These institutions and individuals have the ability to coordinate their behavior, if they choose, and to ensure that capacities are more evenly distributed, and that a higher proportion of the population have a greater capacity to take responsibility for their behavior. The extent to which some groups of individuals are exposed to serious stressors to a significantly greater extent than others, and the extent to which some grow up in resource-poor environments is a result in very important part of political choices we have made, and the circumstance and capacities of individuals are very significantly within the sphere of our control.

We can begin to address inequality in capacities and circumstances of choices in much the same way as we might address other inequalities. For instance, we can ensure that there is an adequate safety net, so that parents are not highly anxious about getting or keeping their jobs. Equally, we can ensure that jobs are adequately paid, so that parents need only work one job. If we do these kinds of things, we ensure that parental stress levels are lower. That’s important, because stress is communicated, advertently or not. Stressed parents have stressed children. Indeed, the stress response begins in utero (O’Donnell *et al.*, 2009): the children of stressed parents have brains preadapted to expect stressors and are hypersensitive to cues for stress. We can ensure that children develop in environments in which they get the opportunity to delay gratification, secure in the knowledge that a reward delayed is not a reward foregone. We can ensure that people are better educated, so that the epistemic conditions on responsibility are better satisfied. We can ensure that people have more opportunities and that the costs of failure are lower. Much of this is familiar, of course: we address responsibility inequalities in much the same ways as we address other inequalities (in fact, social inequality and responsibility inequalities are closely linked: in addressing one, we typically address the other). By doing these things, the individuals and institutions most appropriately held responsible for ill-health would discharge the obligations they have in virtue of being responsible.

Who, precisely, should be responsible for the ways in which the contexts and capacities for choice are distributed is a very difficult question. Making progress on this question will require detailed conceptual and empirical work, for which I have neither the space nor the capacity. Some cases are relatively easy (senior executives at soft drink manufacturers seem to be cases of agents who amply satisfy the control and epistemic conditions; senior politicians, too, are easy cases). Others are much harder (how does one hold voters, an extremely heterogenous group, responsible)? While this is an extremely important question, I cannot address it. I will be content if the arguments given here motivate others to take it seriously enough to carry out the detailed investigation required to assess the issue adequately.

Asking low SES individuals to bear responsibility for adverse health outcomes is asking those with the least capacity to take responsibility to bear it. It is subjecting them to a double dose of unfairness: the unfairness of having to act in unpropitious circumstances with reduced capacities for choice, and the unfairness of being penalized in some way for those choices. We do far better to ask those of us with greater capacities to take responsibility. We bear responsibility, indirectly at least, for *their* health outcomes, because our political and social choices structure the environments which ensures their reduced capacities and their more demanding circumstances. Just as we bear responsibility for how incomes, and opportunities, and statuses, are distributed, so we bear responsibility for how responsibility is distributed.^11^

## Footnotes

1. Sally Haslanger (forthcoming) has recently suggested that work on justice has focused too much on distributive justice, neglecting processes whereby some things come to be seen as valuable in the first place. In addition to expanding work on justice in the way she suggests, I aim to show that we need a broader conception of what gets distributed.

2. While it should be acknowledged that the evidence that obesity causes cancer is mainly correlational, correlational data is (defeasible) evidence for causation. In this case, we have good mechanistic models that make the claim that the correlation is indicative of a causal relation plausible. I thank a reviewer for forcing me to think about this issue more deeply.

3. The basic desert sense is sometimes called the *accountability* sense of responsibility, as opposed to the attributability (or appraisal) and answerability sense. See Shoemaker (2015) for elaboration.

4. As a reviewer for this journal points out, there are philosophers who deny that responsibility requires control. These philosophers instead claim that responsibility requires endorsement or expression of the real self. While it is true that members of this school maintain that agents can be responsible for actions or states of affairs they can’t control (see Frankfurt, 1971 for the classic expression of this view), they accept that there is normally a close link between responsibility, at least in the sense at issue (as opposed to the ‘appraisal’ sense) and control, because we typically express our real selves in the actions we control, and fail to do so in those we do not (see Smith, 2008 for discussion).

5. Further, there is evidence that holding people responsible for their ill-health, whether or not they deserve to be so held, does not in fact produce the consequences that might be hoped for. There appears to be an inverse correlation between willingness to support effective policies and holding people responsible for their health; to that extent, it might actual serve as an obstacle to addressing these challenges (AU).

6. A referee for this journal objects to the claim that healthcare is a domain in which we might have obligations, on the grounds that if resource allocation dilemmas are moral dilemmas, it is easy to obligate others. I am far from confident that we shouldn’t accept that it is easy to obligate others: if you reasonably believe that the £5 I offer you would otherwise be spent in a way that is morally better, you may indeed have an obligation to refuse. Whether you have such an obligation depends on whether the domain in which the money would be spent is appropriately moralized.

7. As a reviewer for this journal points out, these kinds of qualifications are needed because we often do not judge agents blameworthy for risking ill-health. The reviewer gives two examples: firefighters and women who intentionally become pregnant. It is worth noting that some people will judge that agents can be responsible for risking ill-health even in these kinds of circumstances, when the conditions mentioned aren’t satisfied. The firefighter who risks her life to rescue a pet might be judged responsible and thought to be less deserving of treatment in virtue of her action. The woman who has several children and has been warned that she is at high risk of death (and therefore leaving them orphans) if she attempts to carry a child to term might also be judged responsible in the sense at issue.

8. Two reviewers for this journal worries that this claim begs the question against those who hold that responsible choice requires indeterminism. While it is true that libertarians are committed to thinking that there are free choices that cannot be explained, they accept that antecedent factors play a very significant role in explaining how we choose. As they sometimes say, such causes incline without necessitating choice. They therefore expect that the overall pattern of choice will reflect prior causal factors (Kane, 2005). Note that this assumption is required to explain the systematic differences in how people choose, such as the correlation with SES.

9. I owe this objection to a reviewer for this journal.

10. It should be acknowledged that the details of the proposed explanation for the differences in the choices that low and high SES individuals make are somewhat speculative and controversial. However, unless we are prepared to think that such systematic differences are brute facts that cannot be explained, we should acknowledge that some such account is correct. As long as the account explains different choices by reference to social facts that are not themselves chosen by low SES individuals (an extremely plausible suggestion, implementation details aside), we should think that their responsibility is mitigated and that those with more power over these facts are morally and practically better situated to take responsibility for them.

11. I am very grateful to two reviewers and the editors of this journal for helpful comments that enabled me to greatly improve this paper.
